# 4a-Hy­droxy-3,3,6,6-tetra­methyl-9-[6-(3,3,6,6-tetra­methyl-1,8-dioxo-2,3,4,5,6,7,8,9-octa­hydro-1*H*-xanthen-9-yl)pyridin-2-yl]-2,3,4,4a,5,6,7,8,9,9a-deca­hydro-1*H*-xanthene-1,8-dione ethanol hemisolvate hemihydrate

**DOI:** 10.1107/S1600536811008658

**Published:** 2011-03-12

**Authors:** Shaaban K. Mohamed, Antar A. Abdelhamid, Ali N. Khalilov, Atash V. Gurbanov, Seik Weng Ng

**Affiliations:** a School of Biology, Chemistry and Material Science, Manchester Metropolitan University, Manchester, England; b Department of Organic Chemistry, Baku State University, Baku, Azerbaijan; c Department of Chemistry, University of Malaya, 50603 Kuala Lumpur, Malaysia

## Abstract

The pyridine ring in the title compound, C_39_H_47_NO_7_·0.5C_2_H_5_OH·0.5 H_2_O, is connected to one 3,3,6,6-tetra­methyl-1,8-dioxoxanthenyl and one 4a-hy­droxy-3,3,6,6-tetra­methyl-1,8-dioxodeca­hydroxanthenyl substituent in the 2- and 6-positions of the ring. In the former substituent, the six-membered xanthenyl ring adopts a flattened envelope conformation (with the methine C atom as the flap) while in the latter, the six-membered xanthenyl ring adopts a twisted envelope conformation (with the C atom bearing the hy­droxy group representing the flap). The hy­droxy H atom forms an intra­molecular hydrogen bond to the pyridyl N atom. An ethanol solvent mol­ecule is disordered with respect to a water mol­ecule in a 1:1 ratio. The water mol­ecule itself is disordered over two positions of equal occupancy.

## Related literature

For 3,3,6,6-tetra­methyl-9-phenyl-3,4,5,6-tetra­hydro-9*H*-xan­thene-1,8(2*H*,7*H*)-dione, see: Rao *et al.* (2009 ▶); Reddy *et al.* (2009 ▶).
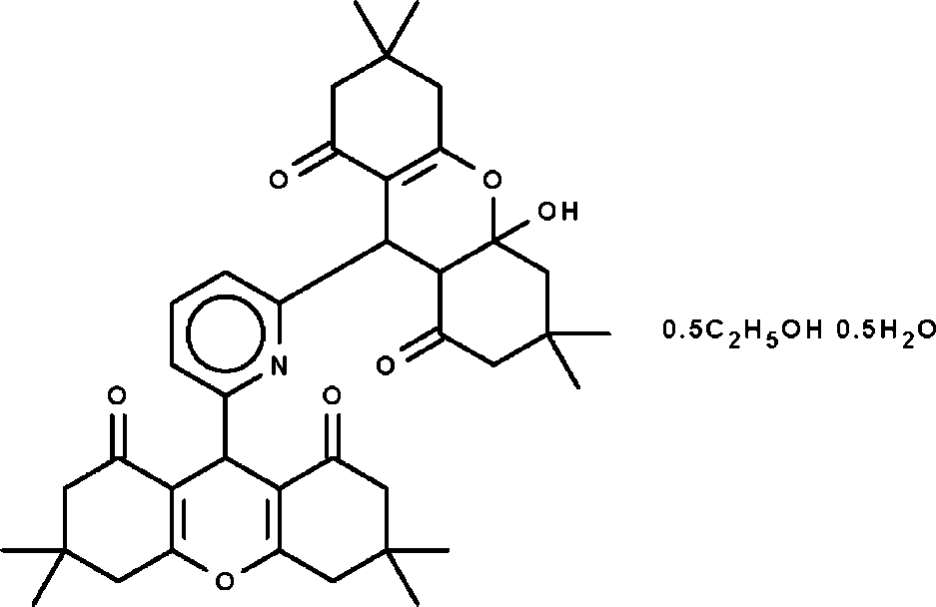


## Experimental

### 

#### Crystal data

C_39_H_47_NO_7_·0.5C_2_H_6_O·0.5H_2_O
*M* *_r_* = 673.82
Monoclinic, 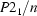

*a* = 14.9739 (5) Å
*b* = 16.3416 (4) Å
*c* = 15.3412 (4) Å
β = 101.113 (3)°
*V* = 3683.56 (18) Å^3^
*Z* = 4
Mo *K*α radiation
μ = 0.08 mm^−1^
*T* = 100 K
0.20 × 0.15 × 0.10 mm

#### Data collection

Agilent SuperNova Dual diffractometer with an Atlas detector
Absorption correction: multi-scan (*CrysAlis PRO*; Agilent, 2010 ▶) *T*_min_ = 0.983, *T*_max_ = 0.992
32212 measured reflections
8332 independent reflections
5349 reflections with *I* > 2σ(*I*)
*R*_int_ = 0.056

#### Refinement

*R*[*F* ^2^ > 2σ(*F* ^2^)] = 0.079
*wR*(*F* ^2^) = 0.247
*S* = 0.99
8332 reflections
472 parameters
33 restraints
H-atom parameters constrained
Δρ_max_ = 0.86 e Å^−3^
Δρ_min_ = −0.43 e Å^−3^

### 

Data collection: *CrysAlis PRO* (Agilent, 2010 ▶); cell refinement: *CrysAlis PRO*; data reduction: *CrysAlis PRO*; program(s) used to solve structure: *SHELXS97* (Sheldrick, 2008 ▶); program(s) used to refine structure: *SHELXL97* (Sheldrick, 2008 ▶); molecular graphics: *X-SEED* (Barbour, 2001 ▶); software used to prepare material for publication: *publCIF* (Westrip, 2010 ▶).

## Supplementary Material

Crystal structure: contains datablocks global, I. DOI: 10.1107/S1600536811008658/im2271sup1.cif

Structure factors: contains datablocks I. DOI: 10.1107/S1600536811008658/im2271Isup2.hkl

Additional supplementary materials:  crystallographic information; 3D view; checkCIF report

## Figures and Tables

**Table 1:** Hydrogen-bond geometry (Å, °)

*D*—H⋯*A*	*D*—H	H⋯*A*	*D*⋯*A*	*D*—H⋯*A*
O6—H6⋯N1	0.84	1.86	2.695 (3)	171

**Fig. 1.**
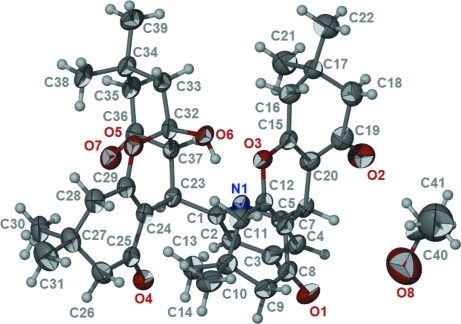
Thermal ellipsoid plot (Barbour, 2001) of C39H47NO7 × 0.5 C2H5OH × 0.5 H2O at the 70% probability level; hydrogen atoms are drawn as spheres of arbitrary radius. The disordered water molecules are not shown.

## supplementary crystallographic information

### Comment

Benzaldehyde (and its derivatives) and dimedone readily condense to form 3,3,6,6-tetramethyl-9-phenyl-3,4,5,6-tetrahydro-9*H*-xanthene-1,8(2*H*,7*H*)-dione (Rao *et al.*, 2009; Reddy *et al.*, 2009), which belongs to the pharmaceutically useful class of xanthenes. An aromatic reactant having two aldehyde groups should therefore furnish a bis-xanthene. In this study, pyridine-2,6-dicarboxaldehyde yielded the expected compound, but one water molecule has added across one of the four carbon-carbon double bonds to yield the title compound (Scheme I, Fig. 1), which was isolated from ethanol as the hemihydrated hemisolvate. The pyridine ring in the title compound, C_39_H_47_NO_7_ × 0.5 C_2_H_5_OH × 0.5 H_2_O, is connected to one tetramethyloctahydroxanthen-1,8-dionyl and one hydroxy-tetramethyldecahydro-xanthen-1,8-dionyl substituent in the 2- and 6-positions of the ring. The hydroxy H atom of the second substituent forms an intramolecular hydrogen bond to the pyridyl N atom.

### Experimental

Amino-*iso*-propanol (20 mmol), pyridine-2,6-dicarboxaldeyde (10 mol) and dimedone (40 mmol) were heated in ethanol (100 ml) for 5 h. The solution was cooled and the brown solid collected and recrystallized from ethanol to give colorless crystals, m.p. 348 K. The synthesis did not yield the expected decahydroacridine derivative (in which the *iso*-propylamino group would replaced the ether oxygen of the xanthene unit).

### Refinement

Carbon- and oxygen-bound H-atoms were placed in calculated positions [C—H 0.95 to 0.98 Å and O–H 0.84 Å; *U*_iso_(H) 1.2 to 1.5*U*_eq_(C,*O*)] and were included in the refinement in the riding model approximation.

The ethanol molecule is statistically disodered with respect to a water molecule As the occupancy refined to a nearly 1:1 ratio, the disordered was interpretated as a hemisolvate hemihydrate. For the ethanol molecule, the carbon–carbon distance was tightly restrained to 1.500±0.005 Å and the carbon–oxygen distance to 1.450±0.005 Å; the carbon···oxygen distance was restrained to 2.30±0.01 Å. The water molecule is disordered over two positions and the occupancies were fixed to 0.25. The temperature factors of the disordered atoms were restrained to be nearly isotropic. The H-atoms of the disordered water components were placed in arbitrary positions.

### Crystal data

**Table:**

C_39_H_47_NO_7_·0.5C_2_H_6_O·0.5H_2_O	*F*(000) = 1448
*M**_r_* = 673.82	*D*_x_ = 1.215 Mg m^−^^3^
Monoclinic, *P*2_1_/*n*	Mo *K*α radiation, λ = 0.71073 Å
Hall symbol: -P 2yn	Cell parameters from 7177 reflections
*a* = 14.9739 (5) Å	θ = 2.5–29.2°
*b* = 16.3416 (4) Å	µ = 0.08 mm^−^^1^
*c* = 15.3412 (4) Å	*T* = 100 K
β = 101.113 (3)°	Block, colorless
*V* = 3683.56 (18) Å^3^	0.20 × 0.15 × 0.10 mm
*Z* = 4	

### Data collection

**Table:**

Agilent SuperNova Dual diffractometer with an Atlas detector	8332 independent reflections
Radiation source: SuperNova (Mo) X-ray Source	5349 reflections with *I* > 2σ(*I*)
Mirror	*R*_int_ = 0.056
Detector resolution: 10.4041 pixels mm^-1^	θ_max_ = 27.5°, θ_min_ = 2.5°
ω scans	*h* = −19→19
Absorption correction: multi-scan (*CrysAlis PRO*; Agilent, 2010)	*k* = −21→21
*T*_min_ = 0.983, *T*_max_ = 0.992	*l* = −19→19
32212 measured reflections	

### Refinement

**Table:**

Refinement on *F*^2^	Primary atom site location: structure-invariant direct methods
Least-squares matrix: full	Secondary atom site location: difference Fourier map
*R*[*F*^2^ > 2σ(*F*^2^)] = 0.079	Hydrogen site location: inferred from neighbouring sites
*wR*(*F*^2^) = 0.247	H-atom parameters constrained
*S* = 0.99	*w* = 1/[σ^2^(*F*_o_^2^) + (0.117*P*)^2^ + 4.4589*P*] where *P* = (*F*_o_^2^ + 2*F*_c_^2^)/3
8332 reflections	(Δ/σ)_max_ = 0.001
472 parameters	Δρ_max_ = 0.86 e Å^−^^3^
33 restraints	Δρ_min_ = −0.43 e Å^−^^3^

### Fractional atomic coordinates and isotropic or equivalent isotropic displacement parameters (Å^2^)

**Table:**

	*x*	*y*	*z*	*U*_iso_*/*U*_eq_	Occ. (<1)
O1	0.75927 (15)	0.77336 (14)	0.87079 (15)	0.0395 (5)	
O2	0.77541 (18)	0.46119 (15)	0.93769 (19)	0.0547 (7)	
O3	0.98264 (13)	0.59879 (12)	0.79452 (13)	0.0308 (5)	
O4	0.60599 (15)	0.70975 (13)	0.48043 (14)	0.0380 (5)	
O5	0.86434 (13)	0.54279 (12)	0.48832 (13)	0.0321 (5)	
O6	0.87367 (14)	0.50643 (13)	0.63467 (12)	0.0326 (5)	
H6	0.8350	0.5344	0.6546	0.049*	
O7	0.64466 (15)	0.46546 (15)	0.38099 (14)	0.0411 (6)	
N1	0.73745 (16)	0.58234 (14)	0.69454 (15)	0.0282 (5)	
C1	0.6656 (2)	0.57624 (17)	0.62681 (18)	0.0282 (6)	
C2	0.5773 (2)	0.58455 (18)	0.6405 (2)	0.0326 (7)	
H2	0.5272	0.5802	0.5922	0.039*	
C3	0.5630 (2)	0.59941 (18)	0.7260 (2)	0.0340 (7)	
H3	0.5029	0.6048	0.7369	0.041*	
C4	0.6369 (2)	0.60629 (18)	0.79493 (19)	0.0312 (6)	
H4	0.6286	0.6169	0.8537	0.037*	
C5	0.7238 (2)	0.59743 (16)	0.77669 (18)	0.0274 (6)	
C6	0.8084 (2)	0.60758 (18)	0.84914 (18)	0.0297 (6)	
H6A	0.7884	0.6177	0.9068	0.036*	
C7	0.86317 (19)	0.68054 (18)	0.82926 (18)	0.0285 (6)	
C8	0.8285 (2)	0.76311 (19)	0.84065 (19)	0.0339 (7)	
C9	0.8824 (2)	0.83457 (19)	0.8171 (2)	0.0376 (7)	
H9A	0.9255	0.8523	0.8710	0.045*	
H9B	0.8402	0.8806	0.7978	0.045*	
C10	0.9359 (2)	0.81661 (19)	0.7437 (2)	0.0345 (7)	
C11	0.9942 (2)	0.74128 (18)	0.7714 (2)	0.0323 (6)	
H11A	1.0212	0.7228	0.7207	0.039*	
H11B	1.0447	0.7562	0.8206	0.039*	
C12	0.9416 (2)	0.67286 (17)	0.80053 (18)	0.0287 (6)	
C13	0.8699 (2)	0.8003 (2)	0.6555 (2)	0.0426 (8)	
H13A	0.8312	0.7533	0.6624	0.064*	
H13B	0.9047	0.7885	0.6090	0.064*	
H13C	0.8317	0.8486	0.6388	0.064*	
C14	0.9966 (3)	0.8890 (2)	0.7313 (3)	0.0466 (8)	
H14A	1.0302	0.8765	0.6841	0.070*	
H14B	1.0397	0.8994	0.7869	0.070*	
H14C	0.9588	0.9377	0.7150	0.070*	
C15	0.9490 (2)	0.53201 (17)	0.83130 (18)	0.0297 (6)	
C16	1.0129 (2)	0.46121 (19)	0.8363 (2)	0.0386 (7)	
H16A	1.0694	0.4744	0.8791	0.046*	
H16B	1.0293	0.4533	0.7774	0.046*	
C17	0.9737 (2)	0.38063 (18)	0.86477 (19)	0.0327 (7)	
C18	0.9207 (3)	0.3984 (2)	0.9381 (2)	0.0445 (8)	
H18A	0.8910	0.3474	0.9525	0.053*	
H18B	0.9638	0.4159	0.9922	0.053*	
C19	0.8487 (2)	0.4640 (2)	0.9131 (2)	0.0422 (8)	
C20	0.8707 (2)	0.53404 (18)	0.8603 (2)	0.0326 (7)	
C21	0.9099 (3)	0.3420 (2)	0.7854 (2)	0.0520 (9)	
H21A	0.8845	0.2912	0.8043	0.078*	
H21B	0.9441	0.3299	0.7385	0.078*	
H21C	0.8604	0.3801	0.7627	0.078*	
C22	1.0509 (3)	0.3204 (2)	0.9000 (3)	0.0536 (9)	
H22A	1.0251	0.2696	0.9184	0.080*	
H22B	1.0918	0.3447	0.9510	0.080*	
H22C	1.0850	0.3084	0.8530	0.080*	
C23	0.68419 (19)	0.55711 (18)	0.53536 (18)	0.0285 (6)	
H23	0.6233	0.5521	0.4952	0.034*	
C24	0.73411 (19)	0.62459 (17)	0.49690 (17)	0.0281 (6)	
C25	0.6849 (2)	0.70053 (18)	0.47057 (18)	0.0309 (6)	
C26	0.7338 (2)	0.76777 (19)	0.4303 (2)	0.0378 (7)	
H26A	0.7685	0.8016	0.4788	0.045*	
H26B	0.6880	0.8035	0.3936	0.045*	
C27	0.7992 (2)	0.7355 (2)	0.3726 (2)	0.0379 (7)	
C28	0.8633 (2)	0.6738 (2)	0.4273 (2)	0.0362 (7)	
H28A	0.8976	0.6448	0.3876	0.043*	
H28B	0.9078	0.7038	0.4721	0.043*	
C29	0.8159 (2)	0.61251 (18)	0.47347 (18)	0.0305 (6)	
C30	0.7455 (2)	0.6935 (2)	0.2894 (2)	0.0445 (8)	
H30A	0.7878	0.6725	0.2534	0.067*	
H30B	0.7041	0.7331	0.2546	0.067*	
H30C	0.7101	0.6481	0.3071	0.067*	
C31	0.8551 (3)	0.8054 (2)	0.3445 (3)	0.0561 (10)	
H31A	0.8959	0.7839	0.3071	0.084*	
H31B	0.8911	0.8313	0.3974	0.084*	
H31C	0.8141	0.8460	0.3108	0.084*	
C32	0.83760 (19)	0.48220 (18)	0.54872 (17)	0.0285 (6)	
C33	0.8842 (2)	0.40358 (18)	0.53108 (18)	0.0309 (6)	
H33A	0.8793	0.3647	0.5794	0.037*	
H33B	0.9496	0.4154	0.5350	0.037*	
C34	0.8488 (2)	0.36018 (19)	0.44159 (19)	0.0327 (7)	
C35	0.7443 (2)	0.35343 (19)	0.42789 (19)	0.0334 (7)	
H35A	0.7202	0.3318	0.3677	0.040*	
H35B	0.7280	0.3143	0.4716	0.040*	
C36	0.70108 (19)	0.43421 (19)	0.43842 (18)	0.0304 (6)	
C37	0.73304 (19)	0.47546 (17)	0.52916 (18)	0.0274 (6)	
H37	0.7160	0.4383	0.5751	0.033*	
C38	0.8758 (2)	0.4055 (2)	0.36336 (19)	0.0386 (7)	
H38A	0.9422	0.4105	0.3731	0.058*	
H38B	0.8483	0.4602	0.3584	0.058*	
H38C	0.8540	0.3749	0.3084	0.058*	
C39	0.8886 (2)	0.2740 (2)	0.4475 (2)	0.0439 (8)	
H39A	0.9551	0.2772	0.4570	0.066*	
H39B	0.8654	0.2446	0.3922	0.066*	
H39C	0.8707	0.2449	0.4974	0.066*	
O8	0.6847 (5)	0.5515 (5)	1.0826 (5)	0.096 (2)	0.50
H8	0.7121	0.5738	1.0460	0.144*	0.50
C40	0.6036 (6)	0.5124 (5)	1.0356 (7)	0.074 (3)	0.50
H40A	0.5750	0.4790	1.0766	0.089*	0.50
H40B	0.6177	0.4765	0.9880	0.089*	0.50
C41	0.5423 (5)	0.5800 (5)	0.9973 (5)	0.064 (2)	0.50
H41A	0.4830	0.5732	1.0144	0.096*	0.50
H41B	0.5345	0.5789	0.9324	0.096*	0.50
H41C	0.5690	0.6324	1.0197	0.096*	0.50
O1W	0.6247 (9)	0.5082 (11)	1.0029 (9)	0.078 (4)	0.25
H1W1	0.6624	0.4918	0.9727	0.117*	0.25
H1W2	0.6507	0.5083	1.0566	0.117*	0.25
O2W	0.7207 (11)	0.5105 (9)	1.1478 (11)	0.091 (4)	0.25
H2W1	0.7566	0.4944	1.1155	0.137*	0.25
H2W2	0.6707	0.5210	1.1144	0.137*	0.25

### Atomic displacement parameters (Å^2^)

**Table:**

	*U*^11^	*U*^22^	*U*^33^	*U*^12^	*U*^13^	*U*^23^
O1	0.0376 (12)	0.0404 (12)	0.0439 (13)	0.0042 (10)	0.0165 (10)	−0.0086 (10)
O2	0.0532 (16)	0.0476 (14)	0.0728 (18)	0.0005 (12)	0.0356 (14)	0.0096 (13)
O3	0.0303 (11)	0.0325 (11)	0.0312 (10)	0.0032 (9)	0.0101 (8)	−0.0008 (8)
O4	0.0400 (13)	0.0392 (12)	0.0359 (12)	0.0082 (10)	0.0104 (10)	0.0027 (9)
O5	0.0295 (11)	0.0369 (11)	0.0309 (10)	0.0003 (9)	0.0087 (8)	−0.0005 (9)
O6	0.0322 (11)	0.0419 (12)	0.0230 (10)	0.0057 (9)	0.0033 (8)	−0.0079 (8)
O7	0.0338 (12)	0.0557 (14)	0.0305 (11)	0.0056 (10)	−0.0020 (9)	−0.0054 (10)
N1	0.0300 (13)	0.0304 (12)	0.0259 (12)	−0.0014 (10)	0.0095 (10)	−0.0014 (9)
C1	0.0305 (15)	0.0270 (14)	0.0289 (14)	0.0005 (12)	0.0107 (12)	0.0019 (11)
C2	0.0323 (15)	0.0357 (16)	0.0304 (15)	0.0005 (13)	0.0079 (12)	0.0008 (12)
C3	0.0291 (15)	0.0375 (16)	0.0386 (16)	0.0042 (13)	0.0148 (13)	−0.0001 (13)
C4	0.0325 (16)	0.0337 (15)	0.0300 (14)	0.0002 (13)	0.0127 (12)	−0.0031 (12)
C5	0.0322 (15)	0.0236 (13)	0.0282 (14)	0.0004 (11)	0.0105 (12)	−0.0022 (11)
C6	0.0318 (15)	0.0341 (15)	0.0255 (13)	0.0005 (12)	0.0118 (12)	−0.0038 (11)
C7	0.0304 (15)	0.0314 (15)	0.0244 (13)	0.0020 (12)	0.0070 (11)	−0.0045 (11)
C8	0.0360 (16)	0.0387 (17)	0.0268 (14)	0.0025 (14)	0.0058 (12)	−0.0078 (12)
C9	0.0413 (18)	0.0320 (16)	0.0399 (17)	0.0016 (14)	0.0084 (14)	−0.0076 (13)
C10	0.0361 (17)	0.0331 (16)	0.0351 (16)	−0.0007 (13)	0.0087 (13)	−0.0029 (13)
C11	0.0288 (15)	0.0347 (16)	0.0334 (15)	−0.0034 (13)	0.0059 (12)	−0.0039 (12)
C12	0.0322 (15)	0.0309 (15)	0.0228 (13)	0.0010 (12)	0.0049 (11)	−0.0026 (11)
C13	0.047 (2)	0.0476 (19)	0.0332 (16)	0.0042 (16)	0.0072 (14)	0.0022 (14)
C14	0.047 (2)	0.0356 (17)	0.062 (2)	−0.0022 (15)	0.0220 (17)	−0.0054 (16)
C15	0.0332 (15)	0.0315 (15)	0.0255 (13)	0.0003 (12)	0.0084 (12)	−0.0034 (11)
C16	0.0405 (18)	0.0355 (17)	0.0430 (17)	0.0058 (14)	0.0159 (14)	0.0005 (14)
C17	0.0374 (16)	0.0311 (15)	0.0286 (14)	0.0028 (13)	0.0041 (12)	−0.0017 (12)
C18	0.050 (2)	0.0440 (19)	0.0396 (18)	−0.0028 (16)	0.0101 (15)	0.0018 (15)
C19	0.0433 (19)	0.0430 (19)	0.0413 (18)	−0.0036 (15)	0.0108 (15)	−0.0030 (15)
C20	0.0329 (16)	0.0305 (15)	0.0364 (16)	0.0007 (13)	0.0118 (13)	−0.0018 (12)
C21	0.064 (2)	0.044 (2)	0.047 (2)	0.0032 (18)	0.0071 (18)	−0.0043 (16)
C22	0.058 (2)	0.048 (2)	0.053 (2)	0.0054 (18)	0.0094 (18)	0.0054 (17)
C23	0.0258 (14)	0.0355 (15)	0.0254 (13)	0.0010 (12)	0.0080 (11)	−0.0003 (11)
C24	0.0298 (15)	0.0315 (15)	0.0233 (13)	−0.0018 (12)	0.0059 (11)	−0.0009 (11)
C25	0.0357 (17)	0.0342 (16)	0.0229 (13)	−0.0003 (13)	0.0055 (12)	−0.0026 (11)
C26	0.0424 (18)	0.0342 (16)	0.0344 (16)	−0.0017 (14)	0.0017 (14)	0.0023 (13)
C27	0.0355 (17)	0.0427 (18)	0.0340 (16)	−0.0074 (14)	0.0034 (13)	0.0069 (13)
C28	0.0330 (16)	0.0424 (18)	0.0330 (15)	−0.0071 (14)	0.0058 (13)	0.0008 (13)
C29	0.0296 (15)	0.0346 (15)	0.0256 (13)	−0.0023 (13)	0.0011 (11)	−0.0004 (12)
C30	0.0446 (19)	0.061 (2)	0.0281 (15)	−0.0014 (17)	0.0065 (14)	0.0048 (15)
C31	0.057 (2)	0.054 (2)	0.058 (2)	−0.0138 (19)	0.0138 (19)	0.0176 (18)
C32	0.0297 (15)	0.0347 (15)	0.0209 (13)	−0.0004 (12)	0.0046 (11)	−0.0006 (11)
C33	0.0315 (15)	0.0360 (16)	0.0248 (13)	0.0033 (13)	0.0042 (11)	−0.0033 (12)
C34	0.0328 (16)	0.0399 (17)	0.0253 (14)	0.0025 (13)	0.0052 (12)	−0.0075 (12)
C35	0.0348 (16)	0.0392 (17)	0.0258 (14)	−0.0024 (13)	0.0051 (12)	−0.0079 (12)
C36	0.0252 (14)	0.0397 (16)	0.0274 (14)	−0.0057 (13)	0.0081 (12)	−0.0025 (12)
C37	0.0281 (14)	0.0316 (14)	0.0243 (13)	0.0001 (12)	0.0092 (11)	0.0000 (11)
C38	0.0388 (17)	0.053 (2)	0.0253 (14)	0.0007 (15)	0.0095 (13)	−0.0090 (13)
C39	0.049 (2)	0.0436 (19)	0.0370 (17)	0.0084 (16)	0.0018 (15)	−0.0129 (14)
O8	0.125 (6)	0.082 (5)	0.095 (5)	0.025 (4)	0.055 (5)	−0.013 (4)
C40	0.081 (7)	0.077 (6)	0.071 (6)	0.019 (5)	0.033 (5)	0.012 (5)
C41	0.080 (5)	0.059 (4)	0.060 (4)	0.014 (4)	0.035 (4)	0.014 (4)
O1W	0.047 (6)	0.141 (9)	0.055 (6)	0.037 (6)	0.034 (5)	0.035 (6)
O2W	0.105 (8)	0.077 (7)	0.091 (8)	−0.021 (6)	0.016 (7)	−0.007 (6)

### Geometric parameters (Å, °)

**Table:**

O1—C8	1.226 (4)	C21—H21C	0.9800
O2—C19	1.228 (4)	C22—H22A	0.9800
O3—C15	1.369 (3)	C22—H22B	0.9800
O3—C12	1.369 (3)	C22—H22C	0.9800
O4—C25	1.230 (4)	C23—C24	1.514 (4)
O5—C29	1.346 (4)	C23—C37	1.533 (4)
O5—C32	1.464 (3)	C23—H23	1.0000
O6—C32	1.383 (3)	C24—C29	1.356 (4)
O6—H6	0.8400	C24—C25	1.460 (4)
O7—C36	1.210 (4)	C25—C26	1.516 (4)
N1—C5	1.338 (3)	C26—C27	1.535 (5)
N1—C1	1.347 (4)	C26—H26A	0.9900
C1—C2	1.385 (4)	C26—H26B	0.9900
C1—C23	1.514 (4)	C27—C31	1.527 (5)
C2—C3	1.391 (4)	C27—C28	1.527 (4)
C2—H2	0.9500	C27—C30	1.533 (5)
C3—C4	1.380 (4)	C28—C29	1.484 (4)
C3—H3	0.9500	C28—H28A	0.9900
C4—C5	1.390 (4)	C28—H28B	0.9900
C4—H4	0.9500	C30—H30A	0.9800
C5—C6	1.525 (4)	C30—H30B	0.9800
C6—C20	1.511 (4)	C30—H30C	0.9800
C6—C7	1.511 (4)	C31—H31A	0.9800
C6—H6A	1.0000	C31—H31B	0.9800
C7—C12	1.337 (4)	C31—H31C	0.9800
C7—C8	1.468 (4)	C32—C33	1.511 (4)
C8—C9	1.502 (5)	C32—C37	1.540 (4)
C9—C10	1.531 (4)	C33—C34	1.545 (4)
C9—H9A	0.9900	C33—H33A	0.9900
C9—H9B	0.9900	C33—H33B	0.9900
C10—C11	1.521 (4)	C34—C39	1.524 (4)
C10—C14	1.526 (5)	C34—C38	1.529 (4)
C10—C13	1.538 (4)	C34—C35	1.543 (4)
C11—C12	1.486 (4)	C35—C36	1.492 (4)
C11—H11A	0.9900	C35—H35A	0.9900
C11—H11B	0.9900	C35—H35B	0.9900
C13—H13A	0.9800	C36—C37	1.538 (4)
C13—H13B	0.9800	C37—H37	1.0000
C13—H13C	0.9800	C38—H38A	0.9800
C14—H14A	0.9800	C38—H38B	0.9800
C14—H14B	0.9800	C38—H38C	0.9800
C14—H14C	0.9800	C39—H39A	0.9800
C15—C20	1.332 (4)	C39—H39B	0.9800
C15—C16	1.495 (4)	C39—H39C	0.9800
C16—C17	1.539 (4)	O8—C40	1.437 (5)
C16—H16A	0.9900	O8—H8	0.8400
C16—H16B	0.9900	C40—C41	1.482 (5)
C17—C18	1.525 (4)	C40—H40A	0.9900
C17—C21	1.531 (5)	C40—H40B	0.9900
C17—C22	1.536 (5)	C41—H41A	0.9800
C18—C19	1.516 (5)	C41—H41B	0.9800
C18—H18A	0.9900	C41—H41C	0.9800
C18—H18B	0.9900	O1W—H1W1	0.8400
C19—C20	1.475 (4)	O1W—H1W2	0.8401
C21—H21A	0.9800	O2W—H2W1	0.8399
C21—H21B	0.9800	O2W—H2W2	0.8399
			
C15—O3—C12	118.1 (2)	H22A—C22—H22C	109.5
C29—O5—C32	118.4 (2)	H22B—C22—H22C	109.5
C32—O6—H6	109.5	C24—C23—C1	113.8 (2)
C5—N1—C1	119.7 (2)	C24—C23—C37	109.4 (2)
N1—C1—C2	121.3 (3)	C1—C23—C37	114.3 (2)
N1—C1—C23	117.9 (2)	C24—C23—H23	106.2
C2—C1—C23	120.8 (3)	C1—C23—H23	106.2
C1—C2—C3	119.1 (3)	C37—C23—H23	106.2
C1—C2—H2	120.5	C29—C24—C25	118.9 (3)
C3—C2—H2	120.5	C29—C24—C23	122.4 (3)
C4—C3—C2	119.3 (3)	C25—C24—C23	117.8 (2)
C4—C3—H3	120.3	O4—C25—C24	121.1 (3)
C2—C3—H3	120.3	O4—C25—C26	120.8 (3)
C3—C4—C5	118.8 (3)	C24—C25—C26	118.2 (3)
C3—C4—H4	120.6	C25—C26—C27	113.4 (3)
C5—C4—H4	120.6	C25—C26—H26A	108.9
N1—C5—C4	121.9 (3)	C27—C26—H26A	108.9
N1—C5—C6	116.8 (2)	C25—C26—H26B	108.9
C4—C5—C6	121.3 (2)	C27—C26—H26B	108.9
C20—C6—C7	107.6 (2)	H26A—C26—H26B	107.7
C20—C6—C5	113.9 (2)	C31—C27—C28	109.2 (3)
C7—C6—C5	110.2 (2)	C31—C27—C30	109.1 (3)
C20—C6—H6A	108.3	C28—C27—C30	109.6 (3)
C7—C6—H6A	108.3	C31—C27—C26	110.6 (3)
C5—C6—H6A	108.3	C28—C27—C26	108.2 (2)
C12—C7—C8	118.6 (3)	C30—C27—C26	110.0 (3)
C12—C7—C6	122.5 (3)	C29—C28—C27	113.6 (3)
C8—C7—C6	118.9 (2)	C29—C28—H28A	108.8
O1—C8—C7	121.0 (3)	C27—C28—H28A	108.8
O1—C8—C9	121.1 (3)	C29—C28—H28B	108.8
C7—C8—C9	117.9 (3)	C27—C28—H28B	108.8
C8—C9—C10	114.2 (2)	H28A—C28—H28B	107.7
C8—C9—H9A	108.7	O5—C29—C24	124.3 (3)
C10—C9—H9A	108.7	O5—C29—C28	111.1 (3)
C8—C9—H9B	108.7	C24—C29—C28	124.6 (3)
C10—C9—H9B	108.7	C27—C30—H30A	109.5
H9A—C9—H9B	107.6	C27—C30—H30B	109.5
C11—C10—C14	109.8 (3)	H30A—C30—H30B	109.5
C11—C10—C9	107.7 (3)	C27—C30—H30C	109.5
C14—C10—C9	110.6 (3)	H30A—C30—H30C	109.5
C11—C10—C13	110.0 (3)	H30B—C30—H30C	109.5
C14—C10—C13	108.8 (3)	C27—C31—H31A	109.5
C9—C10—C13	110.0 (3)	C27—C31—H31B	109.5
C12—C11—C10	112.6 (2)	H31A—C31—H31B	109.5
C12—C11—H11A	109.1	C27—C31—H31C	109.5
C10—C11—H11A	109.1	H31A—C31—H31C	109.5
C12—C11—H11B	109.1	H31B—C31—H31C	109.5
C10—C11—H11B	109.1	O6—C32—O5	107.8 (2)
H11A—C11—H11B	107.8	O6—C32—C33	107.8 (2)
C7—C12—O3	122.7 (3)	O5—C32—C33	105.7 (2)
C7—C12—C11	125.4 (3)	O6—C32—C37	113.7 (2)
O3—C12—C11	112.0 (2)	O5—C32—C37	108.5 (2)
C10—C13—H13A	109.5	C33—C32—C37	113.0 (2)
C10—C13—H13B	109.5	C32—C33—C34	117.3 (2)
H13A—C13—H13B	109.5	C32—C33—H33A	108.0
C10—C13—H13C	109.5	C34—C33—H33A	108.0
H13A—C13—H13C	109.5	C32—C33—H33B	108.0
H13B—C13—H13C	109.5	C34—C33—H33B	108.0
C10—C14—H14A	109.5	H33A—C33—H33B	107.2
C10—C14—H14B	109.5	C39—C34—C38	109.6 (3)
H14A—C14—H14B	109.5	C39—C34—C35	108.4 (3)
C10—C14—H14C	109.5	C38—C34—C35	110.0 (2)
H14A—C14—H14C	109.5	C39—C34—C33	107.9 (2)
H14B—C14—H14C	109.5	C38—C34—C33	112.2 (3)
C20—C15—O3	122.4 (3)	C35—C34—C33	108.6 (2)
C20—C15—C16	126.8 (3)	C36—C35—C34	111.8 (2)
O3—C15—C16	110.8 (2)	C36—C35—H35A	109.3
C15—C16—C17	113.6 (3)	C34—C35—H35A	109.3
C15—C16—H16A	108.8	C36—C35—H35B	109.3
C17—C16—H16A	108.8	C34—C35—H35B	109.3
C15—C16—H16B	108.8	H35A—C35—H35B	107.9
C17—C16—H16B	108.8	O7—C36—C35	123.4 (3)
H16A—C16—H16B	107.7	O7—C36—C37	121.7 (3)
C18—C17—C21	109.4 (3)	C35—C36—C37	114.8 (2)
C18—C17—C22	109.0 (3)	C23—C37—C36	111.6 (2)
C21—C17—C22	109.1 (3)	C23—C37—C32	113.9 (2)
C18—C17—C16	109.1 (3)	C36—C37—C32	109.6 (2)
C21—C17—C16	109.9 (3)	C23—C37—H37	107.1
C22—C17—C16	110.2 (3)	C36—C37—H37	107.1
C19—C18—C17	113.2 (3)	C32—C37—H37	107.1
C19—C18—H18A	108.9	C34—C38—H38A	109.5
C17—C18—H18A	108.9	C34—C38—H38B	109.5
C19—C18—H18B	108.9	H38A—C38—H38B	109.5
C17—C18—H18B	108.9	C34—C38—H38C	109.5
H18A—C18—H18B	107.8	H38A—C38—H38C	109.5
O2—C19—C20	120.3 (3)	H38B—C38—H38C	109.5
O2—C19—C18	121.9 (3)	C34—C39—H39A	109.5
C20—C19—C18	117.7 (3)	C34—C39—H39B	109.5
C15—C20—C19	117.9 (3)	H39A—C39—H39B	109.5
C15—C20—C6	123.1 (3)	C34—C39—H39C	109.5
C19—C20—C6	118.7 (3)	H39A—C39—H39C	109.5
C17—C21—H21A	109.5	H39B—C39—H39C	109.5
C17—C21—H21B	109.5	O8—C40—C41	105.4 (6)
H21A—C21—H21B	109.5	O8—C40—H40A	110.7
C17—C21—H21C	109.5	C41—C40—H40A	110.7
H21A—C21—H21C	109.5	O8—C40—H40B	110.7
H21B—C21—H21C	109.5	C41—C40—H40B	110.7
C17—C22—H22A	109.5	H40A—C40—H40B	108.8
C17—C22—H22B	109.5	H1W1—O1W—H1W2	108.0
H22A—C22—H22B	109.5	H2W1—O2W—H2W2	107.4
C17—C22—H22C	109.5		
			
C5—N1—C1—C2	−0.6 (4)	C5—C6—C20—C15	104.6 (3)
C5—N1—C1—C23	−178.6 (2)	C7—C6—C20—C19	156.1 (3)
N1—C1—C2—C3	−0.1 (4)	C5—C6—C20—C19	−81.4 (3)
C23—C1—C2—C3	177.9 (3)	N1—C1—C23—C24	−66.5 (3)
C1—C2—C3—C4	0.6 (4)	C2—C1—C23—C24	115.5 (3)
C2—C3—C4—C5	−0.5 (4)	N1—C1—C23—C37	60.2 (3)
C1—N1—C5—C4	0.7 (4)	C2—C1—C23—C37	−117.8 (3)
C1—N1—C5—C6	−177.1 (2)	C1—C23—C24—C29	120.8 (3)
C3—C4—C5—N1	−0.1 (4)	C37—C23—C24—C29	−8.4 (4)
C3—C4—C5—C6	177.6 (3)	C1—C23—C24—C25	−69.8 (3)
N1—C5—C6—C20	−58.6 (3)	C37—C23—C24—C25	161.0 (2)
C4—C5—C6—C20	123.5 (3)	C29—C24—C25—O4	170.7 (3)
N1—C5—C6—C7	62.4 (3)	C23—C24—C25—O4	1.0 (4)
C4—C5—C6—C7	−115.4 (3)	C29—C24—C25—C26	−9.0 (4)
C20—C6—C7—C12	18.4 (4)	C23—C24—C25—C26	−178.8 (2)
C5—C6—C7—C12	−106.3 (3)	O4—C25—C26—C27	−144.9 (3)
C20—C6—C7—C8	−162.5 (2)	C24—C25—C26—C27	34.8 (4)
C5—C6—C7—C8	72.8 (3)	C25—C26—C27—C31	−172.5 (3)
C12—C7—C8—O1	−176.5 (3)	C25—C26—C27—C28	−52.9 (3)
C6—C7—C8—O1	4.3 (4)	C25—C26—C27—C30	66.9 (3)
C12—C7—C8—C9	1.5 (4)	C31—C27—C28—C29	168.2 (3)
C6—C7—C8—C9	−177.7 (3)	C30—C27—C28—C29	−72.3 (3)
O1—C8—C9—C10	−152.5 (3)	C26—C27—C28—C29	47.7 (4)
C7—C8—C9—C10	29.5 (4)	C32—O5—C29—C24	−13.0 (4)
C8—C9—C10—C11	−53.7 (3)	C32—O5—C29—C28	167.7 (2)
C8—C9—C10—C14	−173.7 (3)	C25—C24—C29—O5	−175.0 (2)
C8—C9—C10—C13	66.2 (3)	C23—C24—C29—O5	−5.7 (4)
C14—C10—C11—C12	169.5 (3)	C25—C24—C29—C28	4.3 (4)
C9—C10—C11—C12	49.0 (3)	C23—C24—C29—C28	173.6 (3)
C13—C10—C11—C12	−70.8 (3)	C27—C28—C29—O5	154.0 (3)
C8—C7—C12—O3	174.8 (2)	C27—C28—C29—C24	−25.4 (4)
C6—C7—C12—O3	−6.1 (4)	C29—O5—C32—O6	−81.2 (3)
C8—C7—C12—C11	−5.4 (4)	C29—O5—C32—C33	163.7 (2)
C6—C7—C12—C11	173.7 (3)	C29—O5—C32—C37	42.3 (3)
C15—O3—C12—C7	−9.6 (4)	O6—C32—C33—C34	174.6 (2)
C15—O3—C12—C11	170.6 (2)	O5—C32—C33—C34	−70.4 (3)
C10—C11—C12—C7	−21.8 (4)	C37—C32—C33—C34	48.1 (3)
C10—C11—C12—O3	158.0 (2)	C32—C33—C34—C39	−166.4 (3)
C12—O3—C15—C20	10.3 (4)	C32—C33—C34—C38	72.7 (3)
C12—O3—C15—C16	−167.7 (2)	C32—C33—C34—C35	−49.1 (3)
C20—C15—C16—C17	10.2 (5)	C39—C34—C35—C36	169.2 (2)
O3—C15—C16—C17	−171.9 (2)	C38—C34—C35—C36	−70.9 (3)
C15—C16—C17—C18	−40.3 (4)	C33—C34—C35—C36	52.2 (3)
C15—C16—C17—C21	79.7 (3)	C34—C35—C36—O7	122.1 (3)
C15—C16—C17—C22	−160.0 (3)	C34—C35—C36—C37	−58.4 (3)
C21—C17—C18—C19	−66.7 (4)	C24—C23—C37—C36	−85.7 (3)
C22—C17—C18—C19	174.1 (3)	C1—C23—C37—C36	145.3 (2)
C16—C17—C18—C19	53.7 (4)	C24—C23—C37—C32	39.0 (3)
C17—C18—C19—O2	144.4 (3)	C1—C23—C37—C32	−89.9 (3)
C17—C18—C19—C20	−37.8 (4)	O7—C36—C37—C23	0.6 (4)
O3—C15—C20—C19	−169.2 (3)	C35—C36—C37—C23	−178.8 (2)
C16—C15—C20—C19	8.4 (5)	O7—C36—C37—C32	−126.5 (3)
O3—C15—C20—C6	4.9 (5)	C35—C36—C37—C32	54.1 (3)
C16—C15—C20—C6	−177.5 (3)	O6—C32—C37—C23	64.0 (3)
O2—C19—C20—C15	−176.4 (3)	O5—C32—C37—C23	−55.9 (3)
C18—C19—C20—C15	5.7 (4)	C33—C32—C37—C23	−172.8 (2)
O2—C19—C20—C6	9.3 (5)	O6—C32—C37—C36	−170.2 (2)
C18—C19—C20—C6	−168.6 (3)	O5—C32—C37—C36	69.9 (3)
C7—C6—C20—C15	−17.9 (4)	C33—C32—C37—C36	−46.9 (3)

### Hydrogen-bond geometry (Å, °)

**Table:**

*D*—H···*A*	*D*—H	H···*A*	*D*···*A*	*D*—H···*A*
O6—H6···N1	0.84	1.86	2.695 (3)	171
